# Environmental Attributes to Respiratory Diseases of Small Ruminants

**DOI:** 10.1155/2014/853627

**Published:** 2014-03-20

**Authors:** Anu Rahal, Abul Hasan Ahmad, Atul Prakash, Rajesh Mandil, Aruna T. Kumar

**Affiliations:** ^1^Department of Veterinary Pharmacology and Toxicology, Uttar Pradesh Pandit Deen Dayal Upadhyaya Pashu Chikitsa Vigyan Vishwavidyalaya Evam Go-Anusandhan Sansthan (DUVASU), Mathura 281001, India; ^2^Department of Veterinary Pharmacology and Toxicology, Govind Ballabh Pant University of Agriculture & Technology, Pantnagar 263145, India; ^3^Directorate of Information and Publications of Agriculture, KAB-I, New Delhi 110012, India

## Abstract

Respiratory diseases are the major disease crisis in small ruminants. A number of pathogenic microorganisms have been implicated in the development of respiratory disease but the importance of environmental factors in the initiation and progress of disease can never be overemphasized. They irritate the respiratory tree producing stress in the microenvironment causing a decline in the immune status of the small ruminants and thereby assisting bacterial, viral, and parasitic infections to break down the tissue defense barriers. Environmental pollutants cause acute or chronic reactions as they deposit on the alveolar surface which are characterized by inflammation or fibrosis and the formation of transitory or persistent tissue manifestation. Some of the effects of exposures may be immediate, whereas others may not be evident for many decades. Although the disease development can be portrayed as three sets of two-way communications (pathogen-environment, host-environment, and host-pathogen), the interactions are highly variable. Moreover, the environmental scenario is never static; new compounds are introduced daily making a precise evaluation of the disease burden almost impossible. The present review presents a detailed overview of these interactions and the ultimate effect on the respiratory health of sheep and goat.

## 1. Introduction 

Indian livestock sector has emerged as one of the key components of national as well as agricultural growth with an annual contribution of 3.93% (2,41,177 crore) of national GDP and 22.14% share in the agricultural GDP. Today, India ranks first with respect to buffalo, second in cattle and goats, and third in sheep population in comparison to the world livestock population [1]. It also provides self-employment opportunities to almost 6.7% of rural work force. Presently, livestock sector holds a substantial share in fulfillment of human food demand and this share is expected to further get doubled by 2030 [2]. To discharge this increasing demand of livestock products, it is essential that India increases the animal population, improves feed conversion efficiency, implements better reproductive policy, and overall improves the livestock health and productivity, that is, excess use of drugs as food additives, fattening agents, prophylactic antipathogenic drugs, boosters of reproductivity, and so forth. The attempt to increase livestock products (meat, eggs, and milk) production has also resulted in the production, accumulation, and dumping of large amounts of different kinds of wastes or pollutants in the environment all over the world. Aerosolization of microbial pathogens, endotoxins, drug residues, pesticides, offensive odour, and dust particles are all inevitable consequences of the generation and handling of waste material of the food production process, originating from animals. For optimizing livestock productivity, it is mandatory that small ruminant rearers realize that they form the front for identification and prophylaxis of entry of disease-causing agents (pathogens) into production systems [3–5] for a reduction in current on-farm vulnerabilities, upgrading food safety and food security, and enhancing their competence for production of a safer and wholesome product [6].

Broadly, the term “environmental pollution” refers to presence of any agent or a chemical in the environment of an individual which is potentially hazardous to either the environmental or individual's health. As such, environmental pollutants may take many forms: chemicals, organisms, and biological materials, as well as energy in its various forms (e.g., noise, radiation, and heat). The actual number of potential pollutants is therefore incalculable. Less than 1% of these pollutants have been subjected to a detailed appraisal in terms of their toxicity and health risks [7]. Furthermore, environmental conditions are never static; they undergo change over time and rare events may occur which may produce long-term health consequences in the exposed living populations. Such interactions between pathogens, their hosts, and novel environments may alleviate or compound the individual pathological responses, ultimately affecting its viability and contributing to insidious persistence or ultimate destruction of life. A suitable example may be the effect of abiotic factors which include insularity, climate, and volcanism on the prevalence and severity of disease in free-ranging sheep on Hawaii's Island [8].

Respiratory diseases are the major disease crisis in small ruminants [9, 10]. A number of epidemiological surveys have established the presence of the principal respiratory viruses and bacteria in majority of respiratory outbreaks. Repeated attempts have been made to tackle these outbreaks by prior vaccination but only limited success has been achieved. The present review discusses the contributions of environmental factors to initiation and progression of respiratory diseases in small ruminants.

## 2. Respiratory System of Small Ruminants

The respiratory tract of an adult goat comes into contact with approximately 7-8 liters of air per minute, that is, 11,000 liters of air in a day. Thus, the quality of inhaled air has major implications on the respiratory health of the animals. The respiratory system of sheep and goats is quite adaptable against a plethora of air contaminants [11] but disruption of defensive mechanisms to get rid of inhaled material may occur if an individual is exposed to highly concentrated particles in certain situations or if an exposure occurs during strenuous labour. Airborne contaminants may then serve as a primary cause of respiratory disease or can exacerbate a preexisting respiratory conditions or pulmonary disease. Depending on the inhaled substance, acute or chronic reactions occur as particles are deposited on the alveolar surface. Acute reactions are characterized by swelling (oedema) and inflammation [12], while chronic reactions are characterized by connective tissue scarring (fibrosis) and the formation of specific aggregates of immune cells (granulomas) [13]. Some of the effects of exposures may be immediate, whereas others such as lung disease related to asbestos deposits may not present for many decades [14].

## 3. Factors Affecting Development of Diseases

The production of disease in an animal is determined by three basic factors: the host, the pathogen, and the environment [15]. The relationship between these three factors can best be represented in the form of a triangle. It is the balance between these three components that decides the initiation and progress of disease. For initiating disease development, an interaction between a highly virulent pathogen and a susceptible host in a disease favourable environment is required. The environment plays a major role in modulating the virulence of the pathogen [16–18] as well as reducing the host defence [19] and thus increasing the susceptibility of the host. A pathogenic agent can certainly gain entry into the animal body and initiate disease development process but the immune system of host can phagocytise the pathogen (e.g., by secreting chemical factors) and thus check the disease progress. On contrary to this, the host can also influence the environment by alterations in the microclimate requirements for disease production for example, abrasions, wound, malnutrition, path physiological conditions, and immunocompromised status [20]. A thorough consideration of interactions amongst these factors allows assessment of risk for disease outbreaks and intervention to reduce the amount of disease.

The severity of onset of clinical disease in the host is decided chiefly by the pathogenicity of the prevalent population of the pathogen. The term pathogenicity includes both virulence and aggressiveness. The adaptation mechanisms of the pathogen to the altered environmental factors play an important role in determining its survival in the host and the environment as a whole. The reduction in heterozygosity in disease resistance genes of bighorn sheep (*O. canadensis*) populations has been associated with highest lungworm parasite loads [21] as compared to domestic sheep which with a lengthier period of local adaptation and enhanced vigor might have also conferred resistance to common parasitic diseases.* Muellerius *spp. infections also typically do not produce clinical disease in domestic sheep [22] but may be more pathogenic in nonadapted hosts such as bighorn [23, 24] and possibly mouflon [25].

The foremost host factor affecting disease development is the presence of susceptible animals in the population. If the host population is largely susceptible to the pathogen in the vicinity, the disease may have the privilege to get transformed into an epidemic. The key player in determining the susceptibility to any pathogen is the immune status of the animal which, in turn, relies on number of environmental variables for its fluctuations. The preceding immune status of the host is frequently critical in determining the occurrence of disease; for example, low virulence pathogens usually produce clinical disease only in immunocompromised hosts while highly virulent pathogens may show morbidity even in healthy host. Animals whose lungs are already compromised from previous diseases usually fall prey to toxicity by leukotoxins and lipopolysaccharides, both potent toxins that, in high levels, act as chemotactic factors for inflammatory cells and promote inflammation and severe lung damage [26]. In kids, such acute outbreaks can occur with low morbidity rates but high mortality rates.

While lungworm infestations in sheep are quite common, the severity of lung lesions was observed only in sheep regularly exposed to high concentrations of volcanic gases after the eruption of Kīlauea in 2008 which may have contributed to immunocompromised lung health, reduced resistance to parasitic infections, and increased susceptibility for severe inflammatory reactions [8]. Such severity of disease is also observed in conjunction with bacterial and/or viral infections or other stress factors characteristic of bighorn sheep pneumonia complex [27–29].

## 4. Environmental Variables

**Figure 1:**
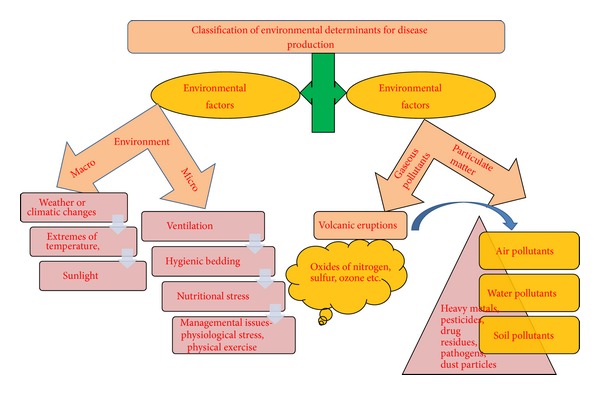
Classification of environmental determinants for disease production.

Environmental variables have conventionally been accepted as the major determinants for disease development (Figure 1). Even the traditional and chemical disease prophylactic and therapeutic control measures employ this concept for manoeuvring the environment to make it less congenial for disease progress. The prevalence of lung disease is unevenly distributed over the world [30] and can be traced down to regional environmental challenges along with other factors such as nutrition. As it is difficult to assess the prevalence, duration, and amount of exposure, the precise risk each environment factor poses is hard to define. Wildlife species of European mouflon sheep (*Ovis gmelini musimon*) translocated to Hawaiian Islands for sport hunting provided a unique opportunity to understand how disease processes may be affected by environmental conditions [8].

## 5. Aerographic Conditions 

The aerographic conditions commonly include the state of atmospheric air in terms of temperature, wind velocity, clouds, precipitation, and volcanic eruptions. The prevailing climatic conditions have a major impact on the survival of the pathogens [31]. An alteration in weather conditions of a geographical area has always witnessed an outburst of infectious diseases and has been labelled as predisposer of disease epidemics. Small ruminants are well adapted to extreme temperatures, with their body hair coats providing insulation against cold and heat [32]. Sheep, in general, are more susceptible than goats to high temperatures and humidity [33]. Any alteration in the environmental temperature affects the incubation period, the life cycle (the time between infection and sporulation), and the contagious period (the time during which the pathogen continues to propagate the infection amongst the population). At higher temperatures the life cycle of the pathogen usually gets speeded up with the result that epidemics develop at a faster rate. Under cooler conditions, the pathogens develop dormancy and the progress of epidemic is slower leading to a decline in incidence as well as severity of disease.

High humidity increases the risk of heat stress at any air temperature. The heat index (temperature + humidity) is considered as a more accurate measure of heat stress (hyperthermia) by veterinarians than temperature alone [34]. Heat stress lowers the natural immune defense of animals, thus, making them more susceptible to disease. An increase in the incidence of pneumonia is a common observation in extremely hot weather [35]. The resistance to parasitic and other opportunistic diseases is also reduced.* P. multocida* often exists as a commensal in the upper respiratory tracts of majority of livestock species and has also been identified as the most frequently isolated bacteria from pneumonic lung [36] but the importance of predisposing factors in the development of pneumonia can never be overestimated.

Moisture also influences outbreak of respiratory diseases caused by microorganisms like bacteria and fungi and nematodes [37]. The influence of rain splash and running water on dispersal of pathogen is also important for explosive nature of the disease [38]. Free water or the collision of raindrops facilitates the dissemination of many fungi and nearly all bacteria. It is a useful adaptation for a pathogen that facilitates dispersal and germination as well as establishment of infection in the host. Pathogens like fungi and nematodes require a latent period for germination of spores and setting up of infection in the host animal. As both these processes are time taking as well as unavoidable for disease initiation, the duration of persistence of favourable climatic conditions has an important influence on infection.

In addition, the dissemination and resulting concentration of the pollutant may vary significantly depending on the prevailing (e.g., meteorological) conditions at that time. Patterns of atmospheric dispersion, for example, change not only in relation to wind speed and direction but also temperature inversion effect and atmospheric stability [39]. Statistically significant relationship was found between incidence of pneumonia as a cause of lamb death and climatic factors such as rainfall, humidity, and intensity and direction of wind [40].

Animal housing is also an important consideration in evaluating the impact of outdoor aerographic conditions on the health of the animals. Animals living indoors are less likely to be affected by rain and thunderstorms but poor ventilation and unhygienic barns are usually associated with severe outbreaks of respiratory diseases. The grazing goats have been reported to show about 2-3-fold higher morbidity as compared to the stall-fed animals [41]. Amongst the indoor factors responsible for microbial pollution the most important is the animal itself and its bedding material. Confinement of circulating air also prevents dissemination of the microbial load and hence facilitates the disease initiation. The moisture content of the bedding material may further assist in production of spores and metabolites of different bacterial and fungal strains resulting in a chronic inflammatory and immunosuppressive response.

## 6. Climate

Climate is the statistical information that expresses the variation of weather at a given place for a specified interval of time. Climate change is likely to directly affect the physiological profile of animal by altering the homeostasis and other thermoregulatory functions and hence its health and productivity. Climate may also influence health of animals indirectly by disturbing the nutritional supply thus, decreasing resistance to diseases and pests. 

*Impact of Climate Change.* Inter-Governmental Panel on Climate Change has projected that global earth temperature will increase by 1.8–4.0°C by the end of this century [42]. This increase in global temperature could potentially cause scarcity of water and food resources and dissemination of infectious diseases and heat-related deaths. The significance of temperature is further promoted in context of temperate regions as compared to the tropics, where temperatures are relatively uniform throughout the year [43]. Further, the subsequent climatic changes are expected to increase the possibility of vector-borne and other diseases and transformation in pattern of disease transmission. The maximum effect of climatic variation on transmission of disease is likely to occur at the lower and upper limits (14–18 and 35–40°C, resp.) of the range of temperature at which the transmission of infection takes place [44]. Rise in temperature and alterations in rainfall pattern will favor the disbursal of vector populations to unforeseen areas (higher altitude or temperate zones) [45]. In the tropics, diurnal oscillations in temperature are greater than the seasonal fluctuations, inducing many pathogens to sporulate by the combination of the decrease in temperature and the increase in humidity at night. The occurrence of Bluetongue in Europe and Rift Valley Fever in goats in East Africa are two well-documented examples of increased vector-borne disease risk in goats associated with climate change [46]. Further, microbial pathogens as well as their vectors may also show sensitivity to factors such as temperature, humidity, rainfall, ground water, wind velocity, and changes in vegetation and are bound to have an impact on emerging and reemerging infections of livestock. In a study conducted in Avikanagar (Rajasthan, India), cold stress along with frost and poor ventilation has been found to predispose lambs to* E. coli*-borne septicemia with major involvement of upper respiratory tract and lungs [47].

## 7. Atmospheric Pollution

Atmospheric pollution remains a major health hazard to all the living species throughout the world and shares about 8-9% of the total disease burden [7], but the risk is higher in developing countries, where poverty, lack of modern technology, and weak environmental legislation further substantiate the risk. The lungs serve as common interface between the animal body and the air environment in its close vicinity. Consequently, the lungs become a frequent dumping site for airborne pollutants. Thousands of environmental toxins and commercial chemicals such as heavy metals and pesticides are now in use, the particles of which may persist in the atmosphere as aerosol, fibres, fumes, mists, or dust. The effects of polluted air on domestic animals principally can either be caused by the indoor environment and by outdoor air pollution. Goats and, to a lesser extent, sheep are reared indoors but their indoor environment is quite comparable with the outdoor air conditions. Therefore, outdoor pollution is considered more important than the indoor pollution. Indoor pollution gains further significance in case of animals kept in overcrowded premises or in poor hygiene or ventilation.

### 7.1. Epidemiology of Atmospheric Pollution

Exposures to pollutants may occur through a number of pathways and exposure processes. Inhalation of environmental pollutants is generally over a considerable period of time and thus usually elicits health issues on chronic basis, but occasional inhalation of solid particles deposited from industrial exhaust on pasture land may directly cause an acute response. The increased incidence of pasture originated disease can be attributed to their short stature due to which they breathe closer to the ground as compared to cattle and hence are more likely to inhale the solid particulates deposited on the pasture. The lesions produced in small ruminants such as sheep and goat due to air pollution are chiefly inflammatory in nature as was observed in 1952 smog disaster (London, UK) that increased respiratory tract hyperresponsiveness and ultimately resulted in respiratory distress (and right-sided heart failure) of cattle that were housed in the city [48] owing to high level of sulphur dioxide. Owing to high solubility sulphur dioxide mainly irritates the anterior air passage characterised by acute bronchiolitis and the accompanying emphysema.

### 7.2. Interplay between Atmospheric Pollution and Health

The relationship between pollution and health is both a multifaceted and conditional process. For pollutants to have an adverse effect on health, susceptible individuals must receive a minimal dose of the pollutant, or its metabolite, over a period sufficient to trigger detectable symptoms. Pollutants rarely occur in isolation; typically they exist in combination [7]. Exposures are therefore not singular rather a mixture of pollutants, often with varied origins, some of which may have additive or synergistic effects [49, 50]. Unravelling the effects of individual pollutants is a herculean challenge that has yet to be adequately resolved in many areas of environmental toxicology. Individual pollutants may be implicated in a wide range of health effects, whereas few diseases can directly be attributed to a single pollutant. Long latent intervals, cumulative exposures, and multiple exposures to different pollutants which might act synergistically all create difficulties in unravelling associations between environmental pollution and health. Health consequences of environmental pollution are thus unpredictable, even for pollutants that are inherently lethal; the ultimate outcome will depend on the coincidence of both the discharge and dispersion processes that determine the rate of appearance and dilution of the pollutant in the environment.

### 7.3. Mechanism of Atmospheric Pollutants

Irrespective of the origin, the ultimate health hazard imposed by all pollutants depends upon their persistence, mobility, biotransformation, and their toxicity profile. The problems associated with the release of persistent pollutants like chlorinated pesticide, DDT (Dichlorodiphenyltrichloroethane), into the environment were highlighted with recognition of the global extent of contamination and a wide-range of environmental and health effects [51]. The signature movement in this regard took long back in 1962 when an American biologist, Rachel Carson, published a book, Silent Spring, and resulted in a large public protest that eventually led to a ban on agricultural use of DDT in the USA in 1972. This book detailed the environmental impacts of the indiscriminate spraying of DDT in the USA and questioned the logic of releasing large amounts of chemicals into the environment without fully understanding their effects on ecology or human health. Similar stories are now around the world in respect to chlorofluorocarbons and other atmospheric pollutants that are accepted as greenhouse gases or scavengers of stratospheric ozone [52] and perhaps also endocrine disruptors [53].

### 7.4. Factors Affecting Pollutants Severity

Mere persistent nature of a pollutant does not endorse the health risk; its presence in a form that is accessible to the lungs is also important to produce respiratory disease. The development of environmentally induced lung disease is a function of the intensity and duration of the exposure as well as the inherent toxicity of the inhaled substance and susceptibility of the host. The physical status of the inhaled substance (solid, fume, or mixture), the particle size, and other physicochemical characteristics (like solubility) principally determine the initial location of disease development. Smaller particles (0.1 to 1.0 *μ*) are more likely to reach the lung alveoli, but airborne particles up to 5 microns in size may also do so. In general, larger particles (10 *μ* or greater) are trapped and removed by the mucus and cilia of the upper respiratory tract. Inorganic mercury is persistent but less toxic and less readily bioavailable than methyl mercury, which gets converted naturally through chemical reactions by microorganisms [54, 55]. Conversely, many solid wastes pose little risk as long as they remain in their original form. The problem arises when their decomposition takes place, either because the decomposition products are inherently more toxic or because they show an increased accessibility to the respiratory system.

Ventilation is often a managemental problem for indoor sheep and goat farming. High level of ammonia is a common finding in the indoor atmosphere of small ruminants. Ammonia is a highly hydrosoluble respiratory toxicant which causes chronic dyspnea and clinical pictures consistent with restrictive lung dysfunction, obstructive lung disease, and bronchial hyperreactivity [56].

### 7.5. Types of Atmospheric Pollutants

Dumping of waste materials of either chemical or biological origin represents a major source of air pollution, though final release into the wider environment may only occur when these materials decompose or break up.

#### 7.5.1. Particulate Matter

Respirable particles of air pollutants and gaseous agents affect different parts of the respiratory tree depending upon their inherent characteristics [57]. For particulate pollutants, particle size is more important while for gasses, relative solubility is important. In a study conducted on Hawaii Island, higher incidence of pathological lesions has been documented in lungworm infested sheep that were exposed to gaseous emissions from Kīlauea Volcano in contrast to lungworm infested sheep not in vicinity of volcanic discharges though latter had significantly more upper respiratory tract inflammation and hyperplasia suggestive of chronic antigenic stimulation, possibly associated with exposure to fine airborne particulates owing to reduced plant ground cover during extended drought conditions [8].

#### 7.5.2. Gaseous Pollutants

Probably, gasses from Kīlauea Volcano such as sulfur dioxide contributed to severity of respiratory disease principally associated with chronic lungworm infections at Mauna Loa. Sulphur dioxide, because it is highly water soluble, initially affects the upper airway, while ozone, with its medium solubility, initially affects the middle airways and nitrogen dioxide, with its low solubility, initially affects the lower airways.

To affect the respiratory tree, the gaseous pollutants must be inhaled in a sufficient volume so that a minimal alveolar concentration is reached. Thereafter, the toxic potency of the pollutant will decide the degree of damage. Different physiological and environmental factors will also exert an influence on the overall toxicity; for example, physiological stress, metabolic acidosis, hypoxia, hypotension, hyponatremia, or hypomagnesaemia will potentiate the toxicity while CNS excitation or hypernatremia will subdue the hazard.

#### 7.5.3. Microbial Contaminants

Bacterial infections in a sheep and goat farm are a common clinical and subclinical finding [58–60]. Some common respiratory commensal bacteria include* Pasteurella *spp. [36],* Staphylococcus *spp.,* Streptococcus pneumoniae* [61],* Arcanobacterium pyogenes*,* Haemophilus *spp., and* Klebsiella pneumonia *while the common mycoplasmas isolated from sheep and goats are* Mycoplasma capricolum *subsp.* capripneumoniae *(a causal agent of caprine contagious pleuropneumonia),* M. mycoides *subsp.* capri *(involved in contagious agalactia syndrome),* M*.* bovis* [62], and* M. ovipneumoniae *[63]. Out of these,* M. ovipneumoniae *is one of the most important mycoplasmas involved in the respiratory diseases of sheep. Combined effects of ammonia and bacterial endotoxins predispose the animals to respiratory infections with viruses and bacteria, both primary pathogenic as well as opportunistic species. Although food producing animals appear to be capable of maintaining a high level of efficient growth in spite of marked degrees of respiratory disease [64], at a certain level of respiratory insufficiency rapid growth can no longer be attained. In that case the production results will be uneconomically. The viral infections also predispose the host to bacterial infection by a direct damage to respiratory clearance mechanisms and lung parenchyma, facilitating translocation of bacteria from the upper respiratory tract and establishment of infection in compromised lung and secondly, by interfering with the immune system's ability to respond to bacterial infection [65, 66].

## 8. Oxidative Stress as Predisposer

Respiratory diseases in sheep and goats are generally an outcome from physiological stress with viral and bacterial infections and adverse weather exposure [67]. Predisposing causes [68] are generally synergistic and include age, stress (comingling, weather, nutritional changes, etc.), and immunological background. Environmental risk factors include climate, ambient temperature, dust particles, stocking density, humidity, ventilation, and shipping distance.

**Figure 2:**
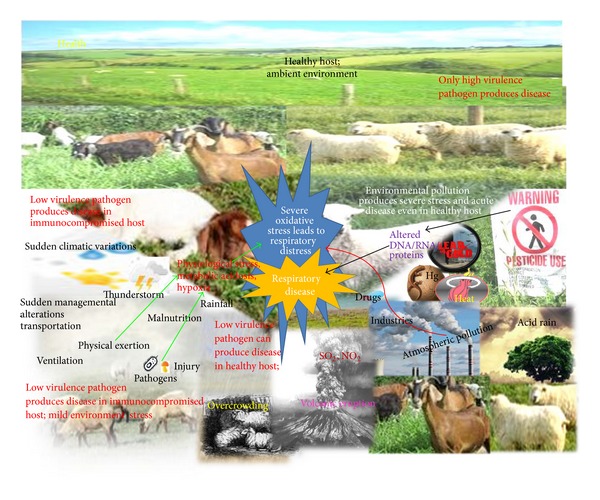
Environmental attributes to oxidative stress leading to initiation and progress of respiratory diseases.

Oxidative stress is a normal physiological phenomenon [69]. Under normal conditions, the physiologically important intracellular levels of reactive oxygen species (ROS) are maintained at a minimal requisite level by various enzyme systems participating in the* in vivo *redox homeostasis. Stress is one of the basic requirements for disease development (Figure 2) [69, 70].

It can have several origins like environmental extremes for example, cold, heat, hypoxia, physical exercise, or malnutrition. Stress can also be categorized on the basis of duration and onset as acute and chronic stress. The stress due to exposure of cold or heat is generally acute and temporary and is released with the removal of cause. Similarly stress due to physical exercises or complete immobilization [71] is also acute in nature but nutritional and environmental stresses usually persist for a longer period of time. Dust, transporting, weaning, handling, mingling with infected animals, overcrowding, dehorning, and castration all add to the onset of disease. Decreasing the number of stress factors associated with a disease is also an important step in prevention. The less an animal is exposed to the stress factors, the more likely it will maintain an integral immune system to defend itself against infectious organisms [72]. Oxidative stress resulting from persistent inflammation due to an inhaled irritant can be the major factor involved in the change of the dynamics of immune responses of the respiratory system. These alterations can create an immunological chaos that could lead to loss of architectural integrity of cells and tissues ultimately leading to chronic conditions or cancers [73, 74].

The significant contribution of predisposing factors in the development of pneumonic lung owing to commensal pasteurella infection is well known [36]. A primary infection with* Mycoplasma ovipneumoniae *is frequently isolated from pneumonic sheep, but it can also be found in the respiratory tracts of healthy animals [75]. Nevertheless, it may predispose sheep to invasion of the lower respiratory tract by other organisms such as the parainfluenza-3 virus and* Mannheimia haemolytica *[76, 77]. Few reports also implicate* Mycoplasma ovipneumoniae *as a cause of severe respiratory disease in goats [78, 79]. Occurrence of clinical respiratory disease due to these pathogens is associated with poor management practices and occur as a consequence of severe stress for example, transportation stress, viral infections (e.g., parainfluenza-3 virus), lung parasites, prior bacterial infections, overcrowded pens, poor housing conditions, sudden environmental changes, and other stressful conditions.

## 9. Prophylactic and Therapeutic Management

The first step in preventing environmentally related lung disease is to recognize the exposure-disease relationship. Then, primary prevention may be accomplished with a reduction, modification, or elimination of the exposure or environment. Other interventions require global approach to prioritize and target environmental modifications with public health policy implications. Educating about the ill effects of air pollution is also an important aspect of prevention of environmentally induced lung disease.

Broad spectrum antibacterial agents may be effective in treating bacterial infections in sheep and goats and may include fluoroquinolones such as enrofloxacin, ciprofloxacin, florfenicol, and ceftiofur along with suitable anti-inflammatory agents [80–83]. While selecting the drug combinations and their respective dosage regimen, drug interaction should to be considered in view of the pathophysiological status of the animal [84]. Several natural feed components have received great attention in the last two decades, and several biological activities showing promising anti-inflammatory, antioxidant, and antiapoptotic-modulatory potential have been identified [85–87]. Plants such as* Ocimum sanctum* have been used for ages to prevent and cure viral infection of man and animals [88].

Interleukin-1beta (IL-1beta) and tumor necrosis factor-alpha (TNF-alpha) have been proven to mediate the development of numerous inflammatory lung diseases [74]. A number of common indigenous plants such as* Cimicifuga racemosa*,* Mimosa pudica*, and so forth have shown excellent anti-inflammatory potential and can be added to regular feeding schedule of small ruminants for prophylaxis [86]. Zinc supplementation has been found to shorten duration of severe pneumonia in human infants. Perhaps, zinc as an adjuvant hastens recovery and reduces antimicrobial resistance [89]. Antioxidant supplements also seem to modulate the impact of ozone and particulates pollutants on lung function [90]. Vitamin C and E may blunt effect of ozone on lung function but do not seem to prevent symptoms.

## 10. Conclusions

Although the disease development can be described as three sets of two-way communications (pathogen-environment, host-environment, and host-pathogen), this is a generalization. All three groups of factors interact in a highly variable manner in any real life scenario, often in nonlinear ways that are difficult to compute and forecast.

Estimating the contribution of environmental pollution to the burden of disease is far from simple. The global atmospheric pollution scenario is too difficult to classify and define completely. Moreover, it is never static; new pollutants are being introduced to the air every day and too little is known about their interactions with respiratory health, or about their levels of exposure, to make reliable toxicity appraisal. These difficulties are more pronounced in developed countries, where disease surveillance, reporting of mortality, environmental monitoring, and population data systems are all relatively well approved. Still precise evaluation of the disease burden is yet worth the endeavour.

The animal biodiversity available in our country is a virtual goldmine of germplasm. Some of the indigenous breed of livestock like Jamunapari goat have unique characteristics of adaptability to adverse agroclimatic conditions, better disease tolerance, feed conversion efficiency, and zero managemental requirements. Therefore, maintain a livestock population that is sustainable in the present everday changing climatic scenario is a challenging task, which would require a change in breeding policy, perpetuating disease resistant and climate adaptable traits, capacity building, and regional and global cooperation.
